# Orthodontic Management of a Patient With Juvenile Idiopathic Arthritis: A Case Report

**DOI:** 10.7759/cureus.66918

**Published:** 2024-08-15

**Authors:** Ahmad Hashridz Ruslan, Mohamad Afiq Farhan Roslan

**Affiliations:** 1 Orthodontics, School of Dental Sciences, Universiti Sains Malaysia Health Campus, Kelantan, MYS; 2 Orthodontics, Edinburgh Dental Institute, The University of Edinburgh, Edinburgh, GBR; 3 Paediatric Dentistry, Edinburgh Dental Institute, The University of Edinburgh, Edinburgh, GBR; 4 Paediatric Dentistry and Orthodontics, Faculty of Dentistry, Universiti Teknologi MARA (UiTM), Sungai Buloh, MYS

**Keywords:** malocclusion, occlusion, treatment, temporomandibular joint, orthodontic, juvenile idiopathic arthritis (jia)

## Abstract

Juvenile idiopathic arthritis (JIA) is a childhood condition marked by joint inflammation, pain, and restricted movement. It often leads to progressive joint damage. Among systemic inflammatory diseases, JIA most commonly affects the temporomandibular joint (TMJ), posing challenges for orthodontic treatment. This case report presents the successful orthodontic treatment of a 15-year-old patient with Class II malocclusion and JIA. The treatment outcome was excellent and remained stable at the one-year follow-up, with no clinical symptoms or radiographic changes in the TMJ observed.

## Introduction

Juvenile idiopathic arthritis (JIA) is a chronic autoimmune inflammatory joint disease that affects children under the age of 16. It is the most common chronic rheumatic disease in children and is characterised by arthritis lasting more than six weeks with an unknown cause [1]. As mentioned by Hariharan et al., the disease presents with various clinical manifestations, including arthritis, spiking fevers, rash, hepatosplenomegaly, lymphadenopathy, and polyserositis [2]. Systemic juvenile idiopathic arthritis (sJIA) is a unique category of JIA characterised by aggressive arthritis, high fevers, rash, and systemic symptoms [3].

Treatment strategies for JIA have evolved significantly with the introduction of conventional therapeutics, new biologics, and methotrexate, leading to improved outcomes. Tumour necrosis factor α (TNFα) inhibitors have shown efficacy in treating JIA [4]. Additionally, tocilizumab has been authorised for use in sJIA and polyarticular JIA [5]. Early physical therapy rehabilitation has been found effective in managing JIA [6]. Surgical management may be necessary for JIA patients with multiple joint involvements [1].

Orthodontic involvement in JIA is crucial, especially in cases where the temporomandibular joint (TMJ) is affected. Early diagnosis of TMJ involvement in JIA is essential, and a pilot study has compared clinical examination, ultrasound (US), and magnetic resonance imaging (MRI) for this purpose, and it was found that although MRI is more accurate for detecting TMJ issues in JIA patients, US can help identify early pathology. This can lead to timely referral for MRI confirmation and appropriate treatment planning. An MRI should be considered after initial US evaluations to confirm diagnoses or improve sensitivity and accuracy in identifying TMJ involvement [7].

Juvenile idiopathic arthritis is a significant health concern in children, requiring a multidisciplinary approach for effective management. Early diagnosis, appropriate medical interventions, physical therapy, and orthodontic care play vital roles in improving outcomes and quality of life for children with JIA. Thus, this case report discusses the relationship between JIA and orthodontics, highlighting the conservative approach of orthodontic treatment in a patient presented with Class II malocclusion.

## Case presentation

A 15-year-old girl presented with the main concern of "protruded and misaligned teeth." She had been diagnosed with JIA, specifically polyarthritis (rheumatoid factor negative), at age three. Her treatment began at the Paediatric Rheumatologic Clinic at the Royal Hospital for Children and Young People in Edinburgh, United Kingdom, after presenting with a large effusion in her left knee, a swollen left ankle, and a stiff left subtalar joint. At that time, she was living on the Isle of Man, and her initial JIA diagnosis was made at Alder Hey Hospital in Liverpool. There was no history of the same problem in her family. Her early treatment included intra-articular steroid injections, subcutaneous methotrexate, and biweekly etanercept, starting in 2014.

In 2015, she returned to Edinburgh and remained stable for two years, leading to the discontinuation of etanercept in early 2017 due to her well-being and difficulties with injections. However, within four months, her symptoms flared, requiring joint injections and alternate-week adalimumab. Throughout her teenage years, she experienced persistent pain in her left ankle, both knees and TMJs due to active disease and mechanical issues.

Her medical history included left ankle arthrodesis and subtalar implant surgery in November 2018, mild synovitis detected by MRI in her left knee in January 2021, and chronic bilateral knee and left ankle pain. An MRI of her TMJs in June 2018 revealed bilateral flattening of the mandibular condyles, more pronounced on the left side, indicating chronic TMJ disease, though no active disease was observed at that time.

During her most recent review in November 2023, she reported discomfort in her right ankle and both knees, although none were swollen or stiff, and no TMJ symptoms were noted.

With the diagnosis of JIA and the persistent pain that she was having, she took adalimumab (Angevita) 40 mg subcutaneous injections (prefilled pen) every other week via homecare, which was commenced in September 2017, and she increased the dose to 40 mg in February 2020. She also had methotrexate as a co-medication (5 mg per oral (PO) weekly), which was commenced in December 2022 with additional naproxen 250 mg twice a day (BD) as required.

**Figure 1:**
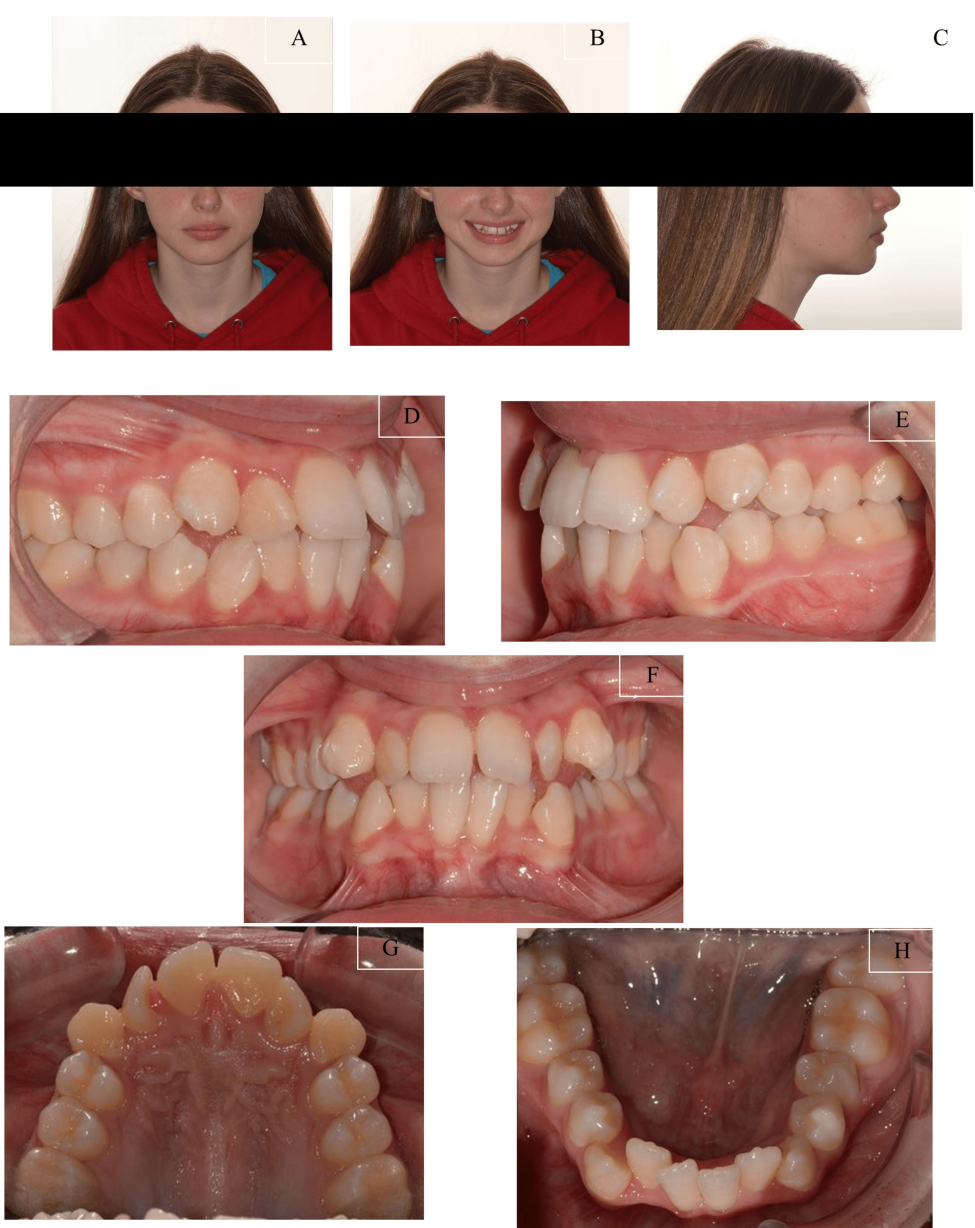
Pre-treatment facial and intraoral photographs of the patient (A) Frontal view of the facial photograph; (B) Smiling view of the facial photograph; (C) Side profile view of the facial photograph; (D) Right view of the intraoral photograph; (E) Frontal view of the intraoral photograph; (F) Left view of the intraoral photograph; (G) Upper occlusal view of the intraoral photograph; (H) Lower occlusal view of the intraoral photograph

The pre-treatment facial photographs revealed a mild convex profile with a mild retrognathic mandible. She displayed an average nasolabial angle and average anterior lower facial height. Her lip was competent, with good incisor exposure at rest and an average smile line. A slight deviation of the nasal septum to the left was also noted (Figures 1A-1C).

**Figure 2:**
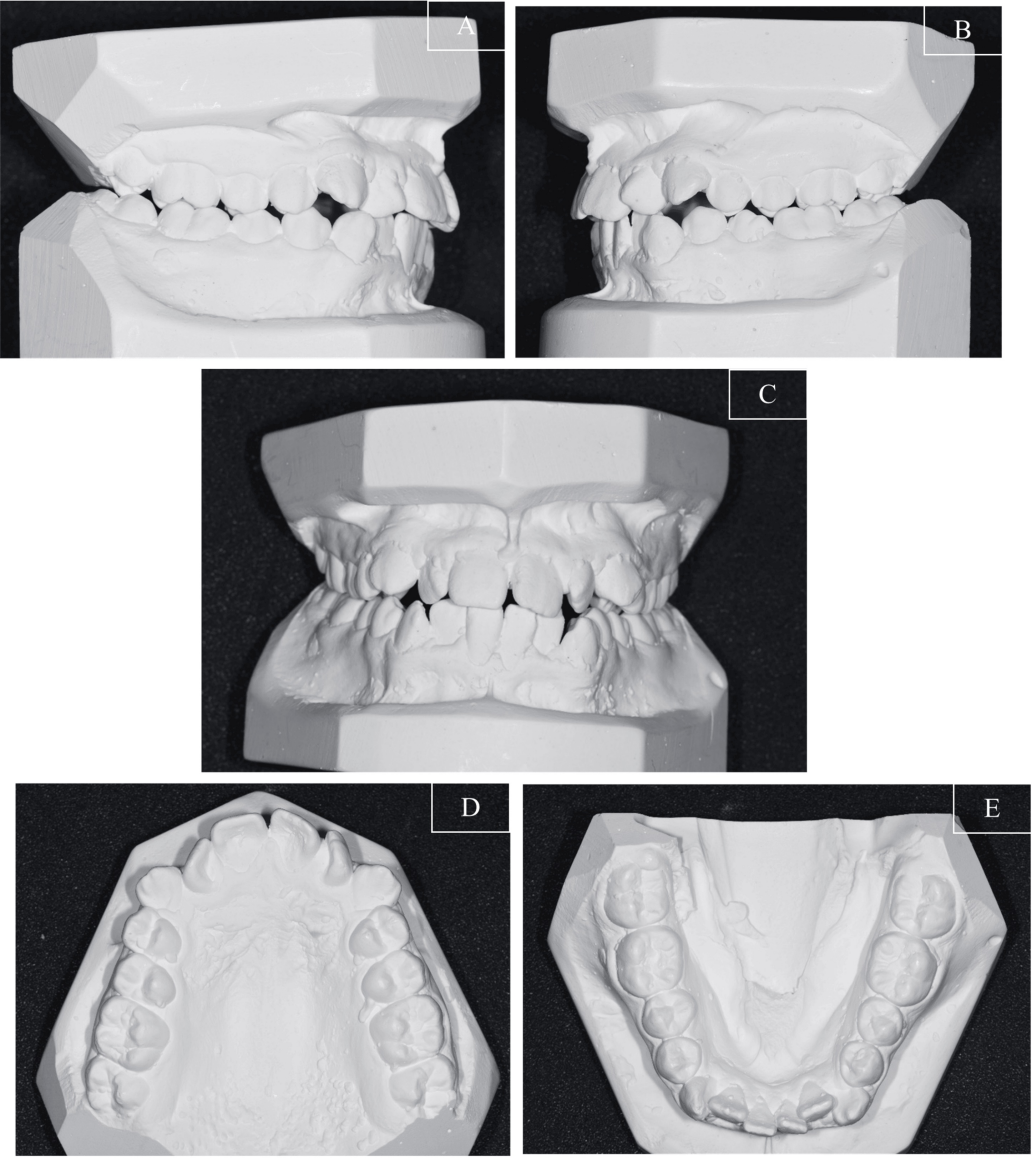
Pre-treatment study model (A) Right view; (B) Left view; (C) Frontal view; (D) Upper occlusal view; (E) Lower occlusal view

The intraoral photographs (Figures 1D-1H) and study models (Figure 2) indicated that she had a Class II Division 1 incisor classification. On the right side, the molars and canines were in a 1/4 unit Class II relationship, while on the left side, the molars and canines were in a 1/2 unit Class III relationship. The overjet measured 5.0 mm, and the overbite measured 2.0 mm clinically.

Both arches exhibited moderate crowding. The maxillary and mandibular dental midlines coincided and were aligned with the facial midline. Additionally, there was a crossbite involving the upper and lower laterals without mandibular displacement. The patient's oral hygiene was slightly suboptimal, with generalised gingivitis present.

**Figure 3:**
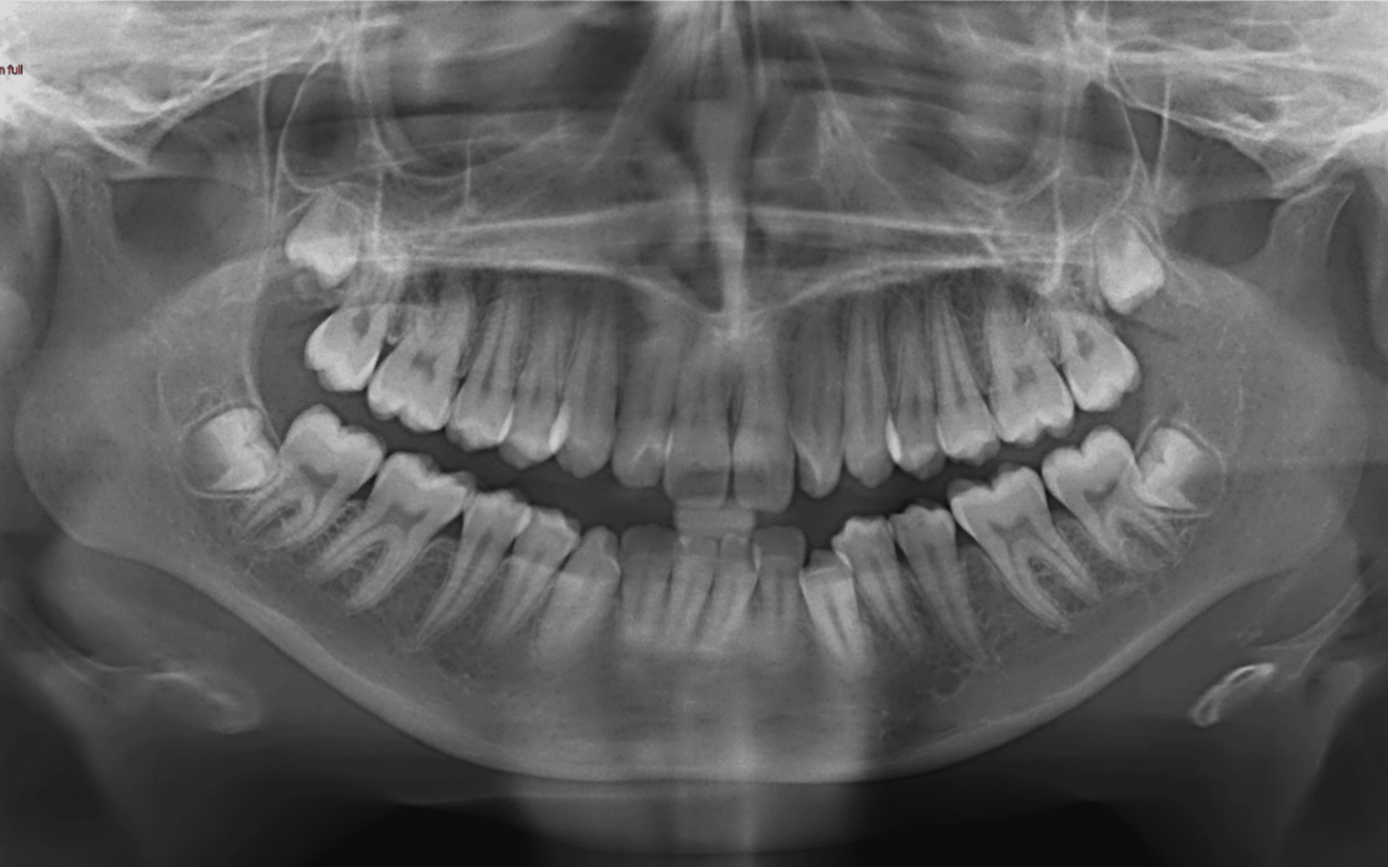
Pre-treatment dental panoramic radiograph

**Figure 4:**
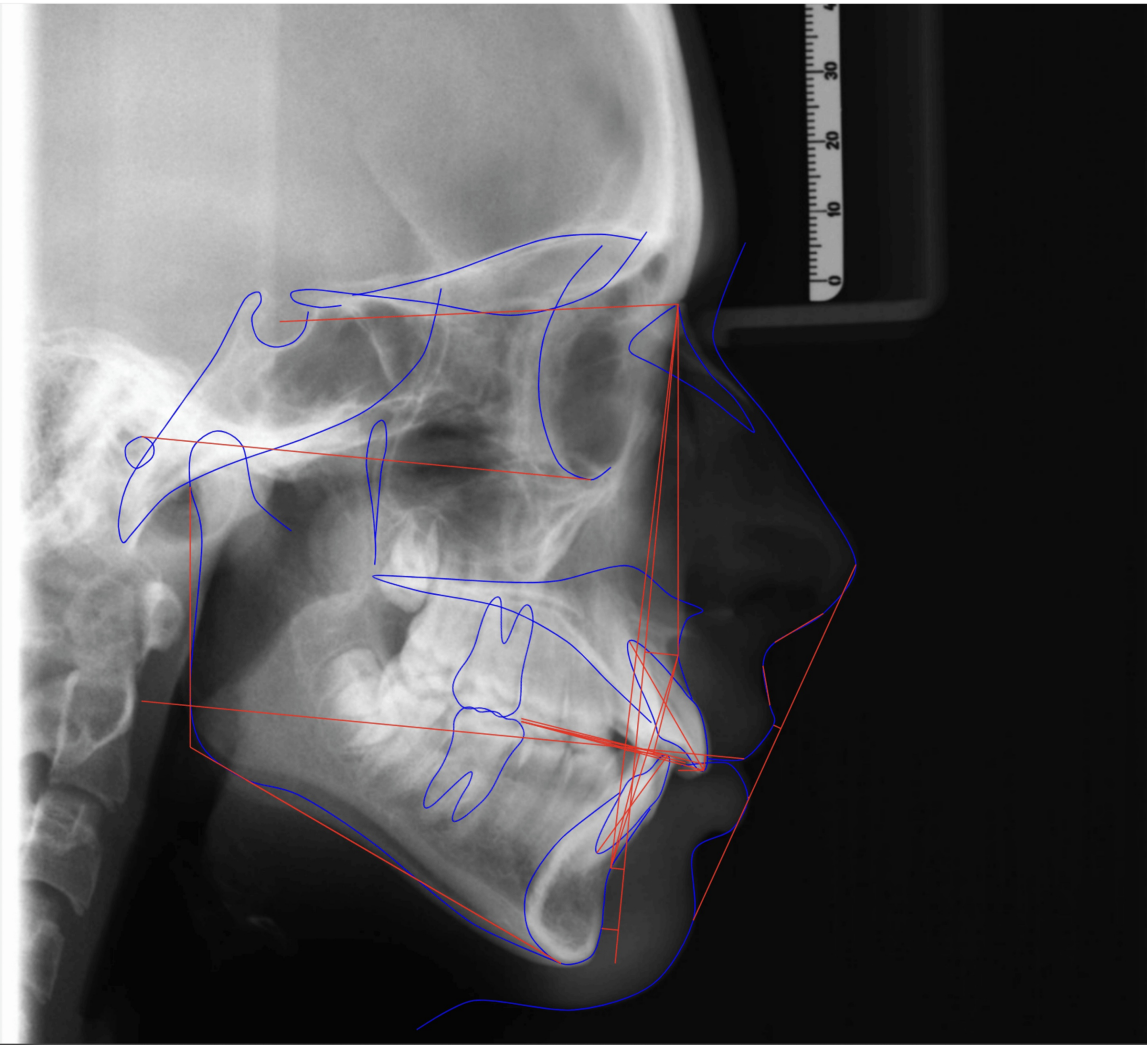
Pre-treatment lateral cephalometry with tracing

The dental panoramic radiograph (Figure 3) revealed normally developing maxillary third molars, thin condyles with signs of resorption, and shallow glenoid activity. The lateral cephalogram (Figure 4) and cephalometric analysis (Table 1) indicated a skeletal Class II relationship (ANB 6.82 degrees). The maxillary and mandibular incisors were proclined (117.46 degrees and 95.74 degrees, respectively), with a decreased interincisal angle. Normal values in the lower anterior facial height and maxillary-mandibular angle (MMPA) suggested that the patient had a normodivergent facial pattern. Additionally, the upper lip was retruded relative to the E line.

**Table 1:** Pre-treatment cephalometric analysis SNA: sella-nasion, A point; SNB: sella-nasion, B point; ANB: A point, B point, and nasion; SN: sella-nasion;

Variable	Norms	T0
SNA	81 +/- 3	87.5
SNB	78 +/- 3	80.68
ANB	3 +/- 2	6.82
Maxila-mandobular plane angle (MMPA)	24 +/- 5	24.78
Face height ratio	55 +/- 2	50.4
SN to maxillary plane	8 +/- 3	7.8
Upper incisor to maxillary plane	114 +/- 7	117.46
Lower incisor to mandibular plane	100 +/- 8	95.74
Interincisal angle	128 +/- 10	114.05
Wits appraisal	-1	6.4
Lower incisor to APo line	3 +/- 0.5	4

Treatment objectives

The patient presented with a Class II Division 1 incisor malocclusion with the molars and canines in a 1/4 unit Class II relationship, while on the left side, the molars and canines were in a 1/2 unit Class III relationship, alongside a skeletal Class II relationship, a mild convex profile with passive lip seal, and moderate crowding in both arches. The treatment goals were to: (1) achieve a functional and normal occlusion with ideal overjet and overbite; (2) alleviate crowding in both the maxillary and mandibular arches; (3) correct the crossbite; (4) establish symmetrical and coordinated dental arches; (5) accept the facial profile appearance; and (6) preserve TMJ health without exacerbating symptoms.

Treatment alternatives

To achieve a proper facial profile and functional, acceptable occlusion, several options were considered: one approach was to camouflage the skeletal discrepancy by extracting the upper first premolars and lower second premolars. This would address the crowding in both arches, normalise the overjet and overbite by retroclining the upper and lower incisor inclinations, and maintain the alignment of the incisors while accepting the skeletal profile. From the cervical vertebral maturation (CVM) staging, the patient would appear to be in stage IV, which is towards the end of the peak pubertal growth stage when she first attended an orthodontic clinic in 2020. Hence, another option would involve modifying growth with a functional appliance and midline expansion to address mandibular retrognathia, using light Class II elastics to correct the molar relationship on the right and light Class III elastics for the left, thereby resolving crowding and improving the maxillary arch form. Rapid maxillary expansion with a Hass-type or Hyrax-type expander and occipital asymmetric headgear were also considered. Given the history of TMJ resorption, the chosen treatment strategy with orthodontic camouflaging and extraction of upper first premolars and lower second premolars is aimed to minimise stress on the TMJ and avoid any adverse effects.

Treatment progress

Upper and lower pre-adjusted edgewise appliances with 0.022” x 0.028” bracket slot size with McLaughlin-Bennet-Trevisi (MBT) prescription was used to correct her malocclusion following the extraction of upper first premolars and lower second premolars. The MBT prescription has increased palatal root torque in the upper incisors (+17° centrals, +10° laterals), which was beneficial in preserving the torque during orthodontic retraction and space closure. Buccal root torque (-7°) in the maxillary canine brackets helped correct the canine root position. The MBT prescription also has increased lingual crown torque (-6°) in the lower incisor brackets, which was beneficial in improving the inclination of the lower incisors following the teeth extraction.

**Figure 5:**
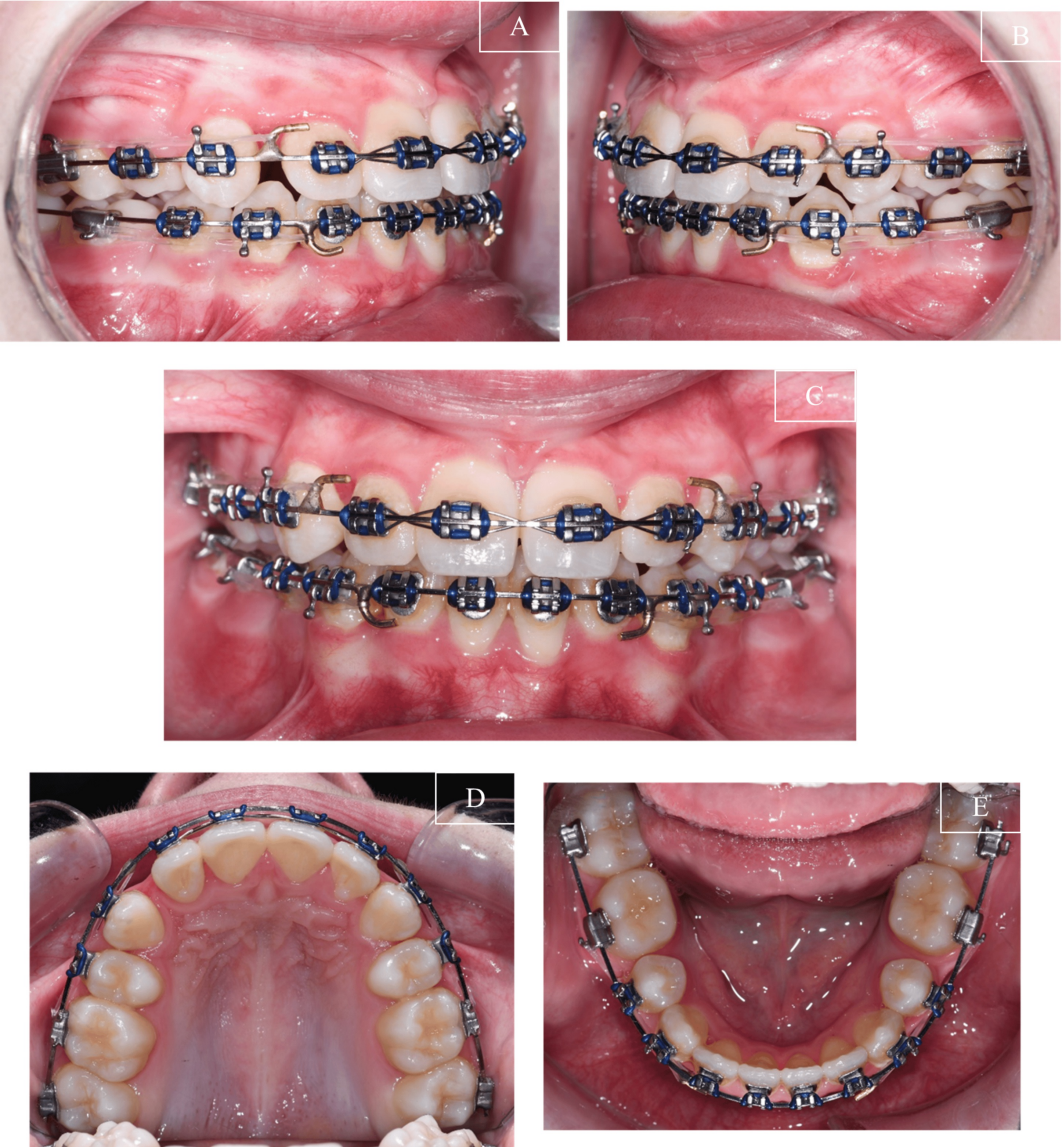
Mid-treatment progress (A) Right view; (B) Left view ; (C) Frontal view; (D) Upper occlusal view; (E) Lower occlusal view

In the upper and lower arches, the initial stage of aligning and levelling was performed, starting with a 0.014” NiTi archwire and progressing to a 0.019 x 0.025 stainless steel working archwire. An elastomeric chain was used to correct the spacing and the molar and canine relationships, as shown in Figure 5. The total treatment duration was 18 months, during which no TMJ symptoms were reported. After treatment, the appliances were removed, and vacuum-formed orthodontic retainers were placed in both the maxillary and mandibular arches. Final records of the patient were obtained immediately after the removal of the appliances.

**Figure 6:**
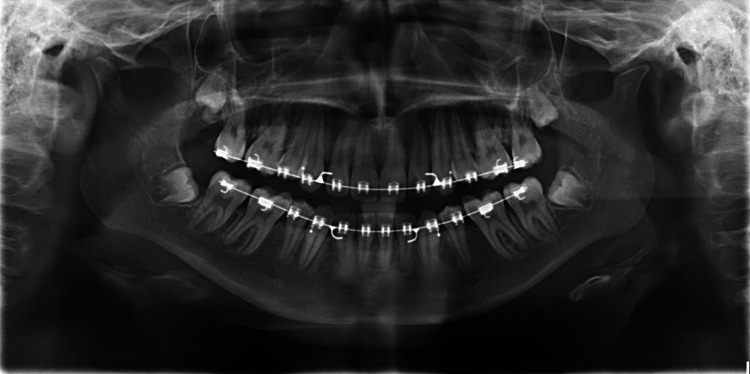
Pre-debond dental panoramic radiograph

From the pre-debond dental panoramic radiograph (Figure 6), it was found that the roots were parallel to each other and there was no evidence of significant root resorption at the apex. The most crucial part of this case, which is the TMJ, also showed that there was no active progression or resorption from the condyle postoperatively.

Treatment results

**Figure 7:**
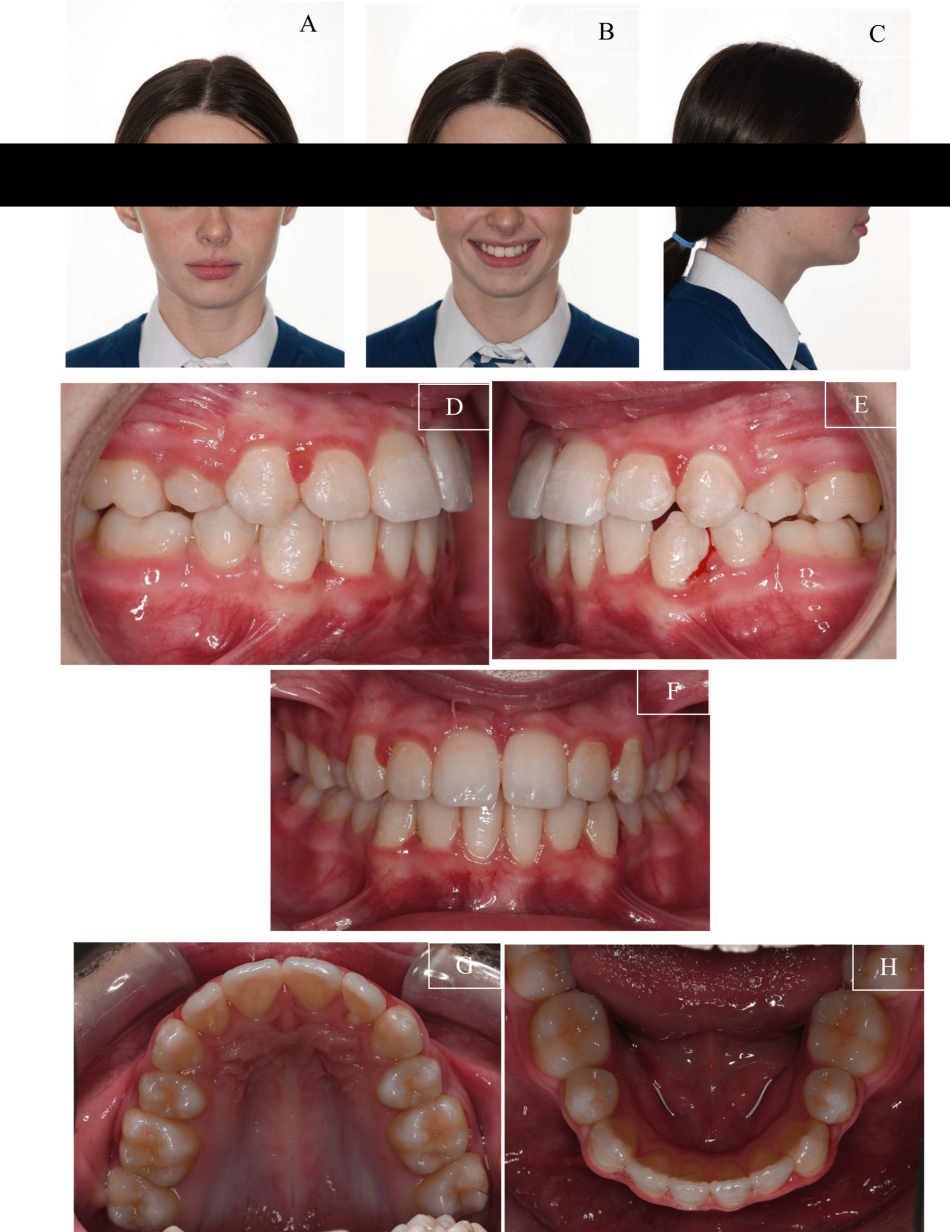
Post-treatment photographs (A) Frontal view of the facial photograph; (B) Smiling view of the facial photograph; (C) Side profile view of the facial photograph; (D) Right view of the intraoral photograph; (E) Frontal view of the intraoral photograph; (F) Left view of the intraoral photograph (G) Upper occlusal view of the intraoral photograph; (H) Lower occlusal view of the intraoral photograph

As shown in Figure 7, the patient's malocclusion was successfully treated over 18 months. Her presenting complaint was addressed, and she was notably pleased with the treatment outcome. An optimal occlusal and aesthetic result was achieved. Her anteroposterior and vertical skeletal patterns were maintained post treatment as observed clinically. The crowding on both the maxillary and mandibular arch was successfully relieved. The incisor inclination was corrected. The crossbite on the upper lateral incisors was also eliminated. The incisor relationship was corrected, and the overbite improved within normal limits. The upper and lower centre lines were coincident with each other and the facial midline. Buccal segment interdigitation was reasonable, and the anticipated settling would further improve occlusal contacts between upper second premolars.

Soft tissues

The soft tissue profile was maintained, and the lips remained competent at the end of treatment.

Iatrogenic findings

Generalised marginal gingivitis was noted on the debond of the fixed appliances, especially on the upper and lower anterior regions. No demineralisation was noted.

Retention

Upper and lower vacuum-formed retainers were provided upon removal of fixed appliances. The prognosis for stability was favourable. A Class I incisor and canine relationship with correct interincisal angle and edge-centroid was achieved. The buccal segment interdigitation was good. Maintenance of the results will depend on her compliance with the retention regime.

## Discussion

Juvenile idiopathic arthritis is a prevalent chronic rheumatic condition with an unclear origin. The aetiologies and how the inflammation becomes chronic are not fully elucidated and are characterised by persistent joint inflammation lasting more than six weeks and onset before the age of 16 [3]. Factors such as autoimmune, genetic, and environmental influences are thought to be significant in triggering the inflammatory response [6, 8]. Chronic inflammation leads to the thickening of the synovium and the breakdown of cartilage and bone, contributing to joint damage. Prognoses vary widely among JIA patients; while many children have mild symptoms and a good prognosis, a notable proportion experience persistent disease activity that can lead to more significant disability [3].

Many children with JIA may develop temporomandibular disorder with the local disturbance in the joint, which can further deteriorate the growth of the TMJ and affect the growth of the mandible. This can result in later complications such as malocclusion and mandibular retrognathia, which are long-term consequences associated with TMJ sequelae [9]. Early detection of TMJ arthritis is crucial to prevent permanent growth disruptions and joint damage. However, detecting temporomandibular joint disorders (TMD) at an early episode can be quite challenging, especially in younger patients when they are not able to communicate effectively in expressing or localising their pain [10]. Thus, timely imaging to detect signs of joint inflammation is important for effective management of the patient.

The treatment of JIA involves using anti-inflammatory and immunomodulatory medications, physical therapy, and potentially surgery, nutritional support, and psychosocial support. The choice of pharmacological treatment is influenced by the specific subtype of JIA, the severity and extent of the disease, any associated conditions, and the family’s acceptance of the treatment plan [4].

Initially, nonsteroidal anti-inflammatory drugs (NSAIDs) are commonly used for symptomatic relief across all JIA subtypes. However, their use has declined with the advent of more aggressive treatments such as methotrexate and biologics [1].

Physical therapy focuses on maintaining a range of motion while minimising stress on the joints. Swimming is often recommended as a beneficial activity. Patients are encouraged to engage in moderate fitness activities, flexibility exercises, and strengthening routines [3].

This case report describes a young woman diagnosed with JIA at age three. Her initial treatment included steroid injections, methotrexate, and etanercept. After moving to Edinburgh in 2015, her condition stabilised, allowing for discontinuation of etanercept in early 2017. However, symptoms flared up, requiring joint injections and adalimumab.

Throughout her teenage years, she experienced persistent pain in her left ankle, both knees and her TMJ. Her medical history included ankle surgery, mild synovitis, and chronic knee and ankle pain. An MRI in 2018 also showed TMJ issues. As of November 2023, she reported discomfort in her right ankle and both knees but no TMJ symptoms. She currently takes adalimumab (40 mg every other week), methotrexate (5 mg weekly), and naproxen as needed.

From the previous study investigating the use of panoramic radiographs to observe TMJ changes, the authors concluded that panoramic radiographs are an efficient technique, showing a prevalence of TMJ abnormalities like those found in other studies using axial computerised tomography. Approximately 39% to 45% of patients exhibited changes on an orthopantomogram, indicating potential diagnoses based on these alterations. However, contrast-enhanced MRI is considered an even more effective and sensitive method for detecting early inflammatory changes and condylar erosions in the TMJ [11]. In this case report, both panoramic radiographs and MRI were taken to detect, monitor, and identify the progression of condylar resorption.

The patient reported no TMJ symptoms. However, many individuals with JIA experience morning joint stiffness, trismus, reduced interincisal opening, limited ability to move the jaw, and clicking or crepitation. About 44% of JIA patients report such subjective symptoms. A study involving 160 JIA patients found that 65% had TMD, with 56% reporting pain and 70% experiencing abnormal mandibular movements. Clinical signs are present in 66% of JIA patients, with restricted mouth opening being the most frequently observed issue [9].

For patients with JIA and dentofacial deformities, the main treatment goal is to restore jaw function and address craniofacial deformities caused by TMJ involvement. Early functional assessment and intervention are crucial due to the potential for limited jaw function and disrupted development of jaw structures. Daily mandibular physiotherapy, including exercises for protrusion, opening, and lateral movements, is recommended to prevent further joint stiffness, facilitate oral hygiene and nutrition, and improve dental treatment outcomes by increasing the range of mandibular movements. This comprehensive approach helps maintain jaw mobility, enhance overall oral health, and optimise the effectiveness of dental treatments [9].

In this case, a skeletal Class II relationship was managed using less invasive orthodontic techniques to camouflage the Class II malocclusion. It is advisable to avoid procedures that may stress the TMJ, such as functional appliances and heavy Class II elastics [12, 13]. However, some suggest that functional appliances might relieve pressure on the condyle and act as "joint protectors," and headgear could be beneficial for children with JIA and moderate mandibular retrognathia [14]. The use of orthognathic surgery in JIA patients is debated, as mandibular repositioning might result in relapse, further condylar resorption, and pain [15]. While some researchers argue that the procedure is safe and provides good occlusal and aesthetic outcomes with minimal relapse, studies supporting this often have limitations, including a broad age range, varying methods, and insufficiently detailed orthodontic treatments [16]. Additionally, these studies typically consist of case observations or series with limited evidence.

The results of the one-year follow-up for our patient showed that the treatment outcomes remained stable, and the results were considered acceptable by both the orthodontic team and the patient, given the challenges associated with the disease.

## Conclusions

There is a lack of definitive evidence on the best orthodontic treatment for patients with JIA and TMJ involvement. The effectiveness of functional appliances and different orthodontic and surgical methods is still debated. Treatment plans should focus on minimising stress on the TMJ to avoid further damage, with recommended mandibular physical therapy. The main goal of orthodontic treatment for this patient is to maintain joint function and improve quality of life. This case report illustrates that orthodontic treatment can effectively manage this condition.
